# Sexuality among elderly patients with dementia: Are we aware of their needs?

**DOI:** 10.1192/j.eurpsy.2022.2086

**Published:** 2022-09-01

**Authors:** L. Santa Marinha, O. Nombora, M. Basto, P. Felgueiras, A. Horta

**Affiliations:** Vila Nova de Gaia Hospital Center, Psychiatry And Mental Health Service, Vila Nova de Gaia, Portugal

**Keywords:** Dementia, sexuality

## Abstract

**Introduction:**

Sexuality is one of the basic needs in human life and its positive effects for the wellbeing are undeniable. People with dementia, despite cognition and functioning impairments, still pursue intimacy as part of their expression of basic human instincts.

**Objectives:**

We aim to address the subject of sexuality among patients with dementia, emphasizing the physiological, environmental and legal barriers.

**Methods:**

We conduct a non-systematic review of recent evidence on dementia and sexuality, using PubMed/Medline database.

**Results:**

People with dementia face several difficulties expressing their sexuality. First, they struggle with physiological barriers to enjoyment of sexuality, such as ageism, apathy and limited free mobility. Secondly, either at home or in long-term care facilities, privacy is usually abolished. For care facilities, the Sexuality Assessment Tool supports the normalization of sexuality and self-audit policies that promote resident rights for privacy and assistance. Moreover, expression of sexuality in elderly can be misinterpreted as disinhibition, leading to unnecessary use of psychotropics to cease these behaviors. Additionally, legal barriers regarding consent arise when a partner loses the ability to consent sexual activity, questioning agreement and mutual desire. The Lichtenberg and Strzepek Decision Tree for Capacity to Participate in Intimate Relationships can be helpful to address this issue.

**Conclusions:**

Sexuality in older people remains neglected in clinical intervention. Besides the urgent need to deconstruct stereotypes, families and staff must be sensitized to understand the changes in expression and perception of sexuality among people with dementia, rather than being indifferent or medicate what can be perceived as disinhibited/distorted expressions of normal needs.

**Disclosure:**

No significant relationships.

